# A Retrospective Study of the Overuse of Extended-Spectrum Antibiotics in Patients With Community-Acquired Pneumonia With Risk for Methicillin-Resistant Staphylococcus aureus and/or Pseudomonas aeruginosa

**DOI:** 10.7759/cureus.31126

**Published:** 2022-11-05

**Authors:** Laura Luu, Ahmed Muhsin

**Affiliations:** 1 Medical School, Oceania University of Medicine, Apia, ASM; 2 Research, Oceania University of Medicine, Texas, USA

**Keywords:** pneumonia, methicillin-resistant staph aureus (mrsa), health care-associated pneumonia (hcap), community-acquired pneumonia (cap), antibiotic overuse

## Abstract

Background

The changing epidemiology of pneumonia is due to aging hosts and the emergence of new pathogens that require ongoing evaluation and updates on antibiotic treatment. Guidelines from the 2019 Infectious Diseases of American Society/American Thoracic Society (IDSA/ATS) provide recommendations for the treatment and management of community-acquired pneumonia (CAP) with risk for methicillin-resistant Staphylococcus aureus (MRSA) and/or Pseudomonas aeruginosa. The objective of this research is to examine patients with CAP with risk for MSRA and/or Pseudomonas aeruginosa to assess the overuse of extended-spectrum antibiotics.

Methods

A retrospective study using a convenience sample of medical records from 118 adult patients with CAP with risk factors for MRSA and/or Pseudomonas was completed from August to December 2019. Descriptive analyses were employed to compute the number and percentages of demographic variables (age, gender), overuse of antibiotics, duration of treatment, and risk factors.

Results

Of 118 patients, 59.3% were males and 77.9% were aged 60 years and older. Seventy-four percent (74%) of patients were prescribed extended-spectrum antibiotics. Most patients (42%) were treated with extended-spectrum antibiotics and did not meet the risk factors based on the 2019 IDSA/ATS recommended guidelines. Twenty percent (20%) were prescribed antibiotics for eight days or more. Patients with known risk factors, including low positive blood (5%) and respiratory (26%) cultures, antibiotic use (25%), and admission to the hospital within 90 days (30%) were identified.

Conclusion

In this sample, the overuse of antibiotics was high. The significant percentage of patients that were over-treated with extended-spectrum antibiotics could lead to adverse outcomes.

## Introduction

Pneumonia is the most common cause of hospital admissions in adults other than women giving birth [1]. About 1 million adults in the United States (US) seek care in a hospital due to pneumonia every year and 50,000 die from this disease [1]. The death rate from pneumonia in the US has had slight improvements since antibiotics became widespread more than half a century ago [1]. Pneumonia is defined as “new lung infiltrates plus clinical evidence that the infiltrate is of an infectious origin, which includes a new onset of fever, purulent sputum, leukocytosis, and decline in oxygenation” [2]. There are several types of pneumonia, which include viral, bacterial, parasite, and fungal. Pneumonia categories are also based on several different methods, including the setting and exposures to infections where pneumonia may develop. Since the publication of the Infectious Diseases Society of America (IDSA)/American Thoracic Society (ATS) guidelines in 2005, pneumonia has been classified as community-acquired pneumonia (CAP), health-care associated pneumonia (HCAP), hospital-acquired pneumonia (HAP), or ventilator-associated pneumonia (VAP) [3]. For this study, we will focus on CAP and HCAP.

HCAP was initially developed to identify patients with risk factors for drug-resistant infections and to prevent inappropriate empiric antimicrobial therapy. Patients with HCAP include those who were hospitalized for two or more days within the preceding 90 days, resided in a nursing home or extended care facility, used home infusion therapy (including antibiotics), had chronic dialysis within 30 days, home wound care, and a history of infection with a multi-drug resistant (MDR) pathogen in a family member [4]. This extensive definition is based on healthcare exposure and risk factors for drug-resistant infections and to prevent inappropriate empiric antimicrobial therapy.

CAP, the most common bacterial pneumonia, is classified as pneumonia that is developed outside the hospital. It is typically attributed to the following bacteria: Streptococcus pneumonia, Haemophilus influenzae, or Legionella species [5]. Comparatively, HCAP is typically infected due to the following potentially antibiotic-resistant bacteria: methicillin-resistant Staphylococcus aureus (MRSA), Pseudomonas aeruginosa, or Acinetobacter species. The recommended treatment for CAP includes, but is not limited to, the following antibiotics: azithromycin, fluoroquinolones, or doxycycline. Extended-spectrum antibiotics are recommended for the treatment of HCAP due to risks for MRSA and/or Pseudomonas aeruginosa. These extended-spectrum antibiotics include vancomycin, linezolid, cefepime, piperacillin-tazobactam, and/or meropenem. Treatment guidelines have been developed to recommend the use of specific antibiotics in the management of pneumonia.

Over the years, the IDSA/ATS has included a panel of experts who have developed clinical practice guidelines and provided evidence-based recommendations to incorporate antimicrobial stewardship and guide medical providers for the treatment and management of pneumonia [6]. Antimicrobial stewardship is a coordinated program that promotes the appropriate use of antibiotics to reduce microbial resistance, decrease the spread of infections caused by multidrug-resistant organisms, and improve patient outcomes [7]. In 2005, HCAP was incorporated by the IDSA/ATS guidelines because of its unique antibiotic treatment due to pathogens that were not susceptible to a standard first-line antibiotic therapy, particularly MRSA and Pseudomonas aeruginosa [3]. Beginning in 2019, the IDSA/ATS guidelines did not identify HCAP categories but provided recommendations for CAP with risks for MRSA and/or Pseudomonas aeruginosa. This change was made because there was a lower risk for MDR pathogens than expected and treatment with extended-spectrum antibiotics did not lead to improved outcomes [4].

Furthermore, the 2019 IDSA/ATS guidelines provided recommendations that HCAP should not be empirically treated with antibiotics to cover MDR bacteria unless there are strong and valid risk factors. These risk factors for MRSA and/or Pseudomonas aeruginosa included: (a) positive respiratory culture, (b) positive blood culture, (c) recent hospitalization, and/or (d) exposure to parenteral antibiotics within 90 days [8]. In addition to these risk factors, local hospital resistance patterns need to be assessed to determine the use of extended-spectrum antibiotics [2]. The strongest risk factors for MRSA and Pseudomonas include known MRSA and Pseudomonas colonization or prior infection [4]. One of the major differences between the current and earlier guidelines updated by IDSA/ATS is that it recommends more microscopic studies of respiratory tract samples in some subgroups of patients to avoid overprescribing antibiotic therapies for drug-resistant bacteria.

Another factor addressed by the 2019 IDSA/ATS guidelines is the de-escalation of antibiotics. Over time, the IDSA/ATS guidelines have been refined to include recommendations about the de-escalation of antibiotics according to the susceptibility of the isolated bacteria. The recommendation stated that the de-escalation of antibiotics should be followed by selecting the narrowest spectrum antibiotic or stopping antibiotics if any non-infectious etiology is established. This would (a) avoid complications of extended-spectrum antibiotic overuse, (b) decrease the length of hospitalizations, and (c) decrease mortality rates [4]. Rello et al reported a decrease in mortality rates when comparing intensive care unit (ICU) mortality rates among those patients that underwent antibiotic de-escalation (18.4%) to those patients who did not undergo de-escalation (43.3%) [9]. This is a key factor for consideration when treating pneumonia.

Antibiotic resistance is another key factor in the treatment and management of CAP who are at risk for MRSA or Pseudomonas aeruginosa. According to a report by the Centers for Disease Control and Prevention (CDC), more than 2.8 million antibiotic-resistant infections occur in the U.S. each year, and more than 35,000 people die as a result [9]. In addition, the CDC has developed the CDC National Action Plan, a five-year plan to reduce antibiotic resistance. This will be accomplished by increasing and enhancing surveillance, adopting evidence-based stewardship strategies, and encouraging innovation and research of new prevention strategies [10]. Methods and strategies to reduce antibiotic resistance are essential to consistently manage the treatment of patients with CAP who are at risk for MRSA and/or Pseudomonas aeruginosa.

Multiple studies have shown the overuse of extended-spectrum antibiotics to treat patients with CAP who are at risk for MRSA and/or Pseudomonas aeruginosa [4,11,12]. The overuse of antibiotics may lead to MDR contributing to severe infection and complications such as (a) antibiotics-related neurological, (b) renal and/or hepatotoxicity, (c) longer hospital stays, and (d) increased mortality. Garnacho-Monterro et al examined the relationship between MDR, inappropriate empiric therapy, and mortality among patients with gram-negative infection [12]. These antibiotics may contribute to other risks such as renal failure in vancomycin use and Clostridium difficile infection in antipseudomonal use. These adverse outcomes and reactions are often associated with increased mortality.

An increased understanding of the use of antibiotics in the treatment of patients with CAP with risks for MRSA and/or Pseudomonas aeruginosa is of paramount importance. Our study hypothesis is that physicians and advanced care providers are empirically overusing extended-spectrum antibiotics to treat patients with CAP who are at risk for MRSA or Pseudomonas aeruginosa. The specific aims of this study are to calculate the prevalence of antibiotic overuse and risk factors associated with the management of CAP with risk factors for MRSA and/or Pseudomonas aeruginosa based on the 2019 ATS/IDSA guidelines in a large urban hospital in Washington State. One goal of the study is to increase medical provider awareness about the use of the 2019 IDSA/ATS guidelines on the treatment and management of pneumonia in efforts to reduce antibiotic resistance, improve patient outcomes, and decrease mortality rates.

## Materials and methods

Data source and study design

Medical records of patients who were admitted with CAP and treated with antibiotics were collected to decide if they met the IDSA/ATS 2019 criteria for needing extended-spectrum antibiotics. Data on antibiotic overuse and associated variables were collected from clinical databases in a large urban hospital in Washington State. All patient-level data were abstracted with the aid of the Pharmacy, Microbiology, and Medical departments in the hospital. Research training required by the hospital research board was completed prior to the abstraction of any records from the hospital records. For this study, all patient and hospital identifiers were de-identified to assure the confidentiality of all patient information.

This research is based on a retrospective study design. The data were collected as a convenience sample of patients who were diagnosed with pneumonia and admitted to a large urban hospital in Washington State. Data were collected for patients who had hospital admissions during the pre-COVID-19 dates, i.e., August 2019 to December 2019.

Inclusion criteria

Inclusion criteria included patients between 18 and 95 years of age who were admitted to a large urban hospital in Washington State. To ensure that we have not included COVID-19 patients in our sample of patients, we restricted the study period to August 2019 to December 2019.

All patients in the study must have had an International Classification of Diseases 10th Revision (ICD-10) diagnosis of community-acquired pneumonia. Second, the patient must have also met the criteria for new-onset pneumonia, i.e., pneumonia symptoms within 21-48 hours. Third, treatment of the antibiotic for pneumonia must have been initiated within 48 hours of admission.

Patients under 18 years of age or older than 95 years of age were excluded from the study. Since the timeline of the study begins at the onset of the cold season in Washington State, August was chosen as the start time for the analysis. The identification of the first cases of coronavirus in January 2020 could potentially impact the outcomes and study results of the study if patients after the end of December were included in the sample. Therefore, all patients admitted after December 2019 were excluded from the study sample. In addition, any patient who had a diagnosis of viral pneumonia, i.e., influenza or COVID pneumonia, acute respiratory distress syndrome, or ventilator-associated pneumonia was excluded from the study.

Statistical analysis

Descriptive statistical analyses were used to describe the antibiotic therapy of patients with CAP with risks for MRSA or Pseudomonas aeruginosa in the study sample. The mean age, as well as the minimum and maximum age values, were computed. Age groups were divided into 10-year intervals and the number of patients and percentages in each age category were computed. In addition, the number of days that extended-spectrum antibiotics were prescribed was determined for each of the categories of days.

Risk factors for MRSA or Pseudomonas aeruginosa were examined. The number and percentages of each of the risk factors, including admission to the hospital or the use of antibiotics in the past 90 days and positive blood and respiratory cultures were determined. The number and percentage of overuse of prescribed extended-spectrum antibiotics without meeting the 2019 IDSA/ATS guideline recommendations were determined.

All analyses of patient data were completed using Microsoft Excel (Microsoft Corporation, Redmond, WA).

## Results

Among the 118 patients who were prescribed antibiotics in a large urban hospital in Washington State, most patients were males (59.3%). Overall, females made up 40.7% of all persons in the study. Most patients (38/118, 32.2%) were in the 60-69 age group, followed by 25.4% in the 80 years and older group. Overall, 92 out of the 118 patients (77.9%) were aged 60 years or older. The mean age of all patients in the study was 67.7 years of age, with a range of 21-92 years of age (Table 1).

**Table 1:** Demographic Characteristics and Number of Days Antibiotics Were Used Among Patients With Community-Acquired Pneumonia (CAP) With Risks for MRSA and/or Pseudomonas aeruginosa - August 2019 to December 2019 (N=118) N = Number of patients in each category; % = Percentage of patients in each category

Characteristics	n	%
Total Sample	118	100%
Mean Age	67.7	
Age Range: Minimum – Maximum	21-92	
Age Groups (in years)		
21-29	5	4.2%
30-39	0	0%
40-49	7	5.9%
50-59	14	11.9%
60-69	38	32.2%
70-79	24	20.3%
80 yrs. and older	30	25.4%
Gender		
Female	48	40.7%
Male	70	59.3%
Number of Days Antibiotics Were Prescribed		
1-3 days	40	33.9%
4-7 days	54	45.8%
8 days and above	24	20.3%

We examined the number of days that all patients in the study were prescribed antibiotics. Among all patients, antibiotics were prescribed over one to nine days. Nearly one-half (54 out of 118, 45.8%) of patients were prescribed antibiotics for four to seven days. In addition, more than one-third (40 out of 118, 33.9%) of all patients were prescribed antibiotics during the past one to three days. Lastly, one-fifth (24 out of 118, 20.3%) of all patients were prescribed antibiotics eight days or more ago (Table 1).

**Figure 1:**
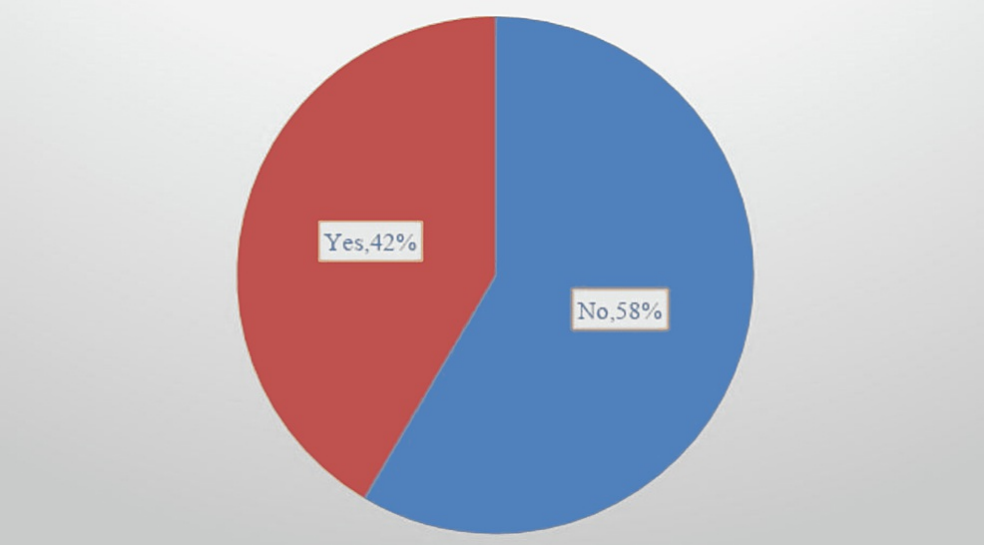
Percent of overuse of antibiotics (1) among all patients in the study - August 2019 to December 2019 (N=118) (1) The overuse of antibiotics is based on the 2019 Infectious Diseases of American Society/American Thoracic Society (IDSA/ATS) Criteria for Treatment of Community-Acquired Pneumonia (CAP) With Risk for MRSA and/or Pseudomonas aeruginosa. MRSA: methicillin-resistant Staphylococcus aureus

**Figure 2:**
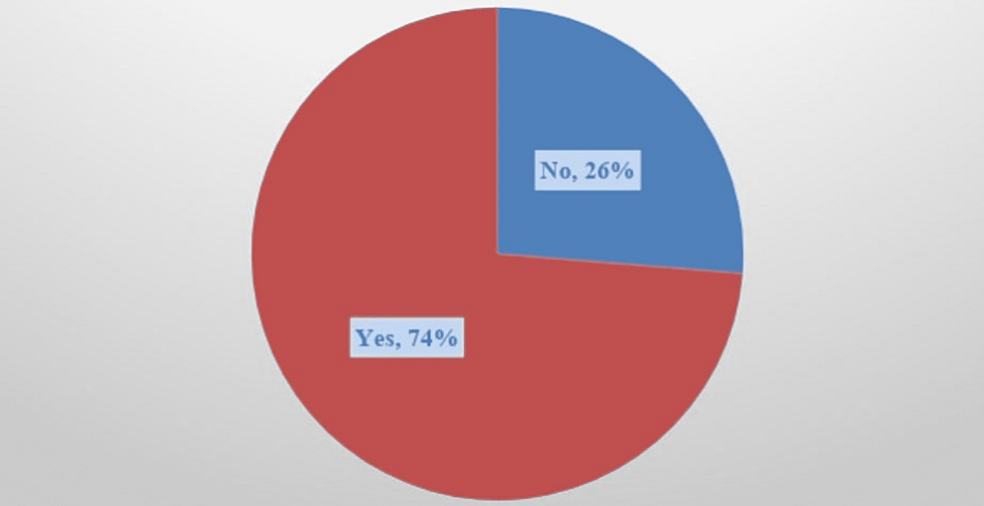
Prescription of extended-spectrum antibiotics (1) used by patients with community-acquired pneumonia (CAP) with risk for MRSA and/or Pseudomonas aeruginosa - August 2019 to December 2019 (N=118) (1) Extended-spectrum antibiotics prescribed included piperacillin-tazobactam, meropenem, vancomycin, linezolid, and cefepime. MRSA: methicillin-resistant Staphylococcus aureus

The antibiotics that were prescribed to all patients were evaluated. Extended-spectrum antibiotics that were prescribed to patients included piperacillin-tazobactam, meropenem, vancomycin, linezolid, and/or cefepime. Overall, 49 out of 118 (42%) patients were prescribed extended-spectrum antibiotics without meeting the 2019 IDSA/ATS guideline recommendations (Figure 1). About three out of every four (74%) of all patients were treated with extended-spectrum antibiotics (Figure 2).

Several risk factors (i.e., recent hospitalizations and exposure to parental antibiotics within 90 days, and positive cultures using blood and respiratory cultures) were examined. For recent hospitalizations, about 35 out of the 118 patients (29.7%) were admitted to the hospital within 90 days. When exposure to parenteral antibiotics was examined, the number of patients who had received antibiotics within 90 days was 28 out of 118 (24.6%). Two types of cultures (blood and respiratory) were collected on all patients. The percentage of positive blood cultures was low, with a prevalence of 5%. More than one-fourth (40 out of 118, 26%) of all respiratory cultures were positive (Table 2).

**Table 2:** Associated risk factors (1) for community-acquired pneumonia (CAP) with risk for MRSA and/or Pseudomonas aeruginosa – August 2019 to December 2019 (N=118) (1)^ ^The associated risk factors are from the 2019 Infectious Diseases of American Society/American Thoracic Society (IDSA/ATS) Criteria for Treatment of Community-Acquired Pneumonia (CAP) With Risk for MRSA and/or Pseudomonas aeruginosa. MRSA: methicillin-resistant Staphylococcus aureus

Characteristics	n	%
Admitted to Hospital Within the Past 90 Days		
No	83	70.3%
Yes	35	29.7%
Antibiotic Use Within the Past 90 days		
No	89	75.4%
Yes	28	24.6%
Results of a Blood Culture		
Negative	112	94.9%
Positive	6	5.1%
Results of a Respiratory Culture		
Negative	87	73.7%
Positive	31	26.3%

## Discussion

This study estimates the prevalence of antibiotic overuse in patients with CAP with risk for MRSA and/or Pseudomonas aeruginosa in a large urban community hospital in Washington State. The study is essential because it highlights the characteristics of persons who were prescribed extended-spectrum antibiotics in a community hospital, as well as the lack of adherence to accepted guidelines for the use of these antibiotics. Overall, the study provides important information to better understand the prescription of antibiotic therapy in patients with CAP with risk for MRSA and/or Pseudomonas aeruginosa in community hospitals.

Most (45.8%) patients were prescribed antibiotic therapy for four to seven days. One out of five (20.3%) patients were prescribed antibiotics eight days or more ago. The recommended duration of antimicrobial treatment for patients with CAP is a minimum of five to seven days and seven days in patients suspected or proven to have Pseudomonas or MRSA infections [13]. In this study, 20% of the patients were prescribed for a longer duration (eight or more days) for use of antibiotics than recommended by the 2019 IDSA/ATS guidelines. The concern is that prolongation of unnecessary antibiotics use may lead to patients acquiring antibiotic-resistant organisms. There is evidence that shorter durations of antibiotics can reduce adverse effects such as toxicity, allergy, and resistance [14].

About three-fourths (74%) of all patients received extended-spectrum antibiotics. Of all patients, 42% were prescribed extended-spectrum antibiotics without meeting the risk factors outlined by the 2019 IDSA/ATS guidelines, which is consistent with other research study’s findings of overuse of antibiotics, i.e., ICU-acquired Pneumonia Study Group and Garnacho-Montero et al. [11,12]. The ICU-acquired Pneumonia Study Group [11], a prospective multicenter study, demonstrated a link between inappropriate empiric therapy and increased mortality. The study by Garnacho-Montero et al. found that mortality occurred in 33.6% of patients who were prescribed initial appropriate antibiotic therapy [12]. Patients treated with inappropriate first antimicrobial treatment were significantly more likely to die during their hospitalizations than patients treated with appropriate initial antimicrobial therapy [15]. Overall, these studies reported that the overuse of antibiotics may lead to increased mortality. For future research, it would be interesting to study mortality rates and the overuse of extended-spectrum antibiotics to treat CAP with risks for MRSA and Pseudomonas aeruginosa.

In this research study, selected risk factors (i.e., recent hospitalizations and exposure to parental antibiotics within 90 days and positive blood and respiratory cultures) were examined. The percentage of positive blood cultures was 5%. More than one-fourth (26%) of all respiratory cultures were positive, about 30% were admitted to the hospital within 90 days and 25% received antibiotics within 90 days. The number of patients who received antibiotics or were admitted to the hospital within 90 days was 25% and 30%, respectively. When examining the validated risk factors defined by the 2019 IDSA/ATS guidelines, this study had low percentages of patients with positive blood and respiratory cultures. These low positive rates suggest that there is not a strong valid need for extended-spectrum antibiotics to treat CAP with MRSA and/or Pseudomonas aeruginosa.

Due to the significant extended-spectrum antibiotics overuse, the antimicrobial stewardship program at this large urban hospital may help guide clinicians to improve antibiotic prescribing and combat antibiotic resistance. The HCAP concept was removed and greater than 20% of hospitalized patients with pneumonia are not specifically addressed by the current guideline recommendations [16]. Since the failure to identify risk factors to treat HCAP appears to be the most common cause in the administration of inappropriate antimicrobial therapy to hospitalized patients, it is recommended that standardized approaches be implemented in local hospitals. This would include the use of a utilization order set to prescribe appropriate antibiotics for CAP with a risk for MRSA and/or Pseudomonas aeruginosa. The standardized order set would require healthcare providers to screen patients for healthcare-associated infection risk factors and treat them with recommended antimicrobials as recommended by the 2019 IDSA/ATS guidelines. In addition, acceptance of the CDC National Action 2020-2025 plan to reduce antibiotic resistance by increasing and enhancing surveillance, adopting evidence-based strategies, and encouraging research of new prevention strategies to control and improve the way antibiotics could be used [10].

The incidence of CAP with risk for MRSA and/or Pseudomonas aeruginosa is low but particularly important in the management of pneumonia. Vardakas KZ et al reported that the estimated incidence of CAP with risk for MRSA and/or Pseudomonas aeruginosa was 0.51 to 0.64 cases per 100,000 [17]. An international multicenter study indicated that the overall prevalence of confirmed CAP with risk for MRSA and/or Pseudomonas aeruginosa was 3% in a sample of patients with microbiology testing [18]. These studies reported low CAP with risk for MRSA and/or Pseudomonas aeruginosa rates and the initiation of extended-spectrum antibiotics for MRSA and Pseudomonas coverage. This suggests a need for reconsideration of the use of the assessment of the patient’s risks as recommended by 2019 IDSA/ATS guidelines.

There are some limitations of this study. First, this study is a retrospective study based on a convenience sample. Because this is a convenience sample, there is a potential for bias in the sample population. We did not employ any sampling or stratification methods to reduce bias in our patient sample selection. Second, the study’s time period was short with small sample size. The peak of the onset of pneumonia typically occurs in the fall and winter seasons. However, the researcher selected the period prior to January 2020 (onset of COVID-19 cases) to exclude any cases of COVID-19 viral pneumonia. The time period of the study was restricted from August 2019 to December 2019. In addition, the sample size was limited to the number of patient records that were available during the selected time frame. Therefore, the sample may not be representative of the entire population. Third, the data for this study was from a single center and may not be applicable to other hospitals. For example, hospitals caring for patients with low risks for HCAP would not expect to see similar rates of inappropriate antibiotic use. Lastly, the researcher limited the study to patients with identified bacterial pneumonia, excluding other causes. These findings are not applicable to patients with nonbacterial causes of pneumonia such as viral pneumonia. There are several strengths of this study. This is one of the first studies to examine the characteristics of patients and associated risk factors among those who are at risk for MRSA and Pseudomonas aeruginosa and treated in a large urban hospital in Washington State. This study will allow us to gain a better understanding of clinical practices followed to treat inpatient CAP with extended-spectrum antibiotics.

## Conclusions

The results of the current study are consistent with the current literature, which shows that patients who were over-prescribed extended-spectrum antibiotics for CAP with a risk for MRSA and/or Pseudomonas still exist. Despite established guidelines, most patients who did not meet the risks for factors for MRSA and/or Pseudomonas received extended-spectrum antibiotics in treating CAP and were prescribed extended-spectrum antibiotics. This is a stark indication that more training is needed about the treatment of patients and adherence to recommended CAP guidelines. This is valuable information because it provides awareness for providers to not overprescribe antibiotics and prolong the duration of antibiotics as recommended by using the 2019 IDSA/ATS guidelines in this world of antibiotic resistance.
